# Dihydropyrimidine dehydrogenase predicts survival and response to interferon-α in hepatocellular carcinoma

**DOI:** 10.1038/s41419-017-0098-0

**Published:** 2018-01-22

**Authors:** Wei-Ping Zhu, Ze-Yang Liu, Yi-Ming Zhao, Xi-Gan He, Qi Pan, Ning Zhang, Jia-Min Zhou, Long-Rong Wang, Miao Wang, Di-Hua Zhan, De-Ning Ma, Lu Wang

**Affiliations:** Department of Hepatic Surgery, Fudan University Shanghai Cancer Center, Shanghai Medical College, Fudan University, Shanghai, 200032 PR China

## Abstract

Metastasis and recurrence contribute to poor prognosis of hepatocellular carcinoma (HCC). Recently, we reported that interferon-α (IFN-α) can suppress metastasis of HCC; however, the underlying mechanism has not been fully described. In this study, we demonstrated that expression of dihydropyrimidine dehydrogenase (DPYD), a pyrimidine catabolic enzyme, was dose-dependently downregulated by IFN-α in HCC tissues from nude mice. Notably, DPYD expression was found to be significantly increased in HCC cell lines with higher metastatic potentials compared with their controls. Moreover, upregulation of DPYD in HCC cells could promote in vitro migration, invasion, and in vivo lung metastasis, and inducing changes characteristic of epithelial-mesenchymal transition (EMT). In contrast, knockdown of DPYD inhibited these processes. Mechanistically, DPYD functioned as a positive regulator of EMT in HCC by targeting the p38/NF-κB/Snail1 pathway. Clinically, tissue microarray analysis showed that high DPYD expression was positively associated with aggressive tumor characteristics, including larger tumor size, tumor recurrence, and advanced tumor node metastasis (TNM) stage, and independently correlated with poorer overall survival times after curative resection. HCC patients with low DPYD expression have better response to IFN-α therapy. Taken together, our findings elucidate that IFN-α could downregulate DPYD expression to inhibit EMT and HCC metastasis, and suggest that DPYD might be a potential prognostic biomarker and a therapeutic target for HCC.

## Introduction

Hepatocellular carcinoma (HCC) is the fifth most common malignancy worldwide, and nowadays rises to the second commonest leading cause of cancer death^1^. The poor prognosis of HCC is mainly attributed to metastasis and recurrence^2,3^. Thus, exploring a therapeutic strategy that can effectively inhibit the metastasis and recurrence of HCC becomes urgently needed. In our previous study, we found that interferon-α (IFN-α) could inhibit tumor metastasis in nude mice bearing human HCC xenografts^4^, and this effectiveness was also validated by our clinical trials^5,6^. IFN-α has been shown to be a promising drug for HCC^7,8^. Unfortunately, some HCC patients are not sensitive to IFN-α therapy. Hence, a better understanding of the antitumor mechanism of IFN-α would provide clinical benefit.

Dihydropyrimidine dehydrogenase (DPYD) is a rate-limiting enzyme of pyrimidine metabolism, which plays an initial and rate-limiting role in uracil and thymidine catabolism. Increasing studies reported that upregulation of DPYD in some human tumors, such as bladder cancer^9^, breast cancer^10^, colorectal cancer^11^, and gastric cancer^12^, was predictive for poor patient prognosis. Recent studies revealed that DPYD was a critical regulator for transcriptional drivers of epithelial–mesenchymal transition (EMT)^13^, a program correlated with the acquisition of metastatic tumor characteristics^14,15^. Although there is increasing evidence to a link between DPYD and tumor, little is known about the role of DPYD in HCC progression.

Here, we identified DPYD as a therapeutic target of IFN-α, which can dose-dependently be downregulated by IFN-α treatment in mice bearing human HCC xenografts. Our data evidenced that DPYD activity is highly elevated in human HCCs, where it promotes metastasis through a mechanism that involves EMT. Moreover, we also confirmed that the p38/NF-κB/Snail1 signaling by which DPYD regulates EMT and facilitates HCC progression. In this study, we identified a metabolic signature related to IFN-α therapy, and further elucidated the mechanism of DPYD in promoting HCC metastasis.

## Results

### Identification of DPYD as a therapeutic target of IFN-α

**Fig. 1:**
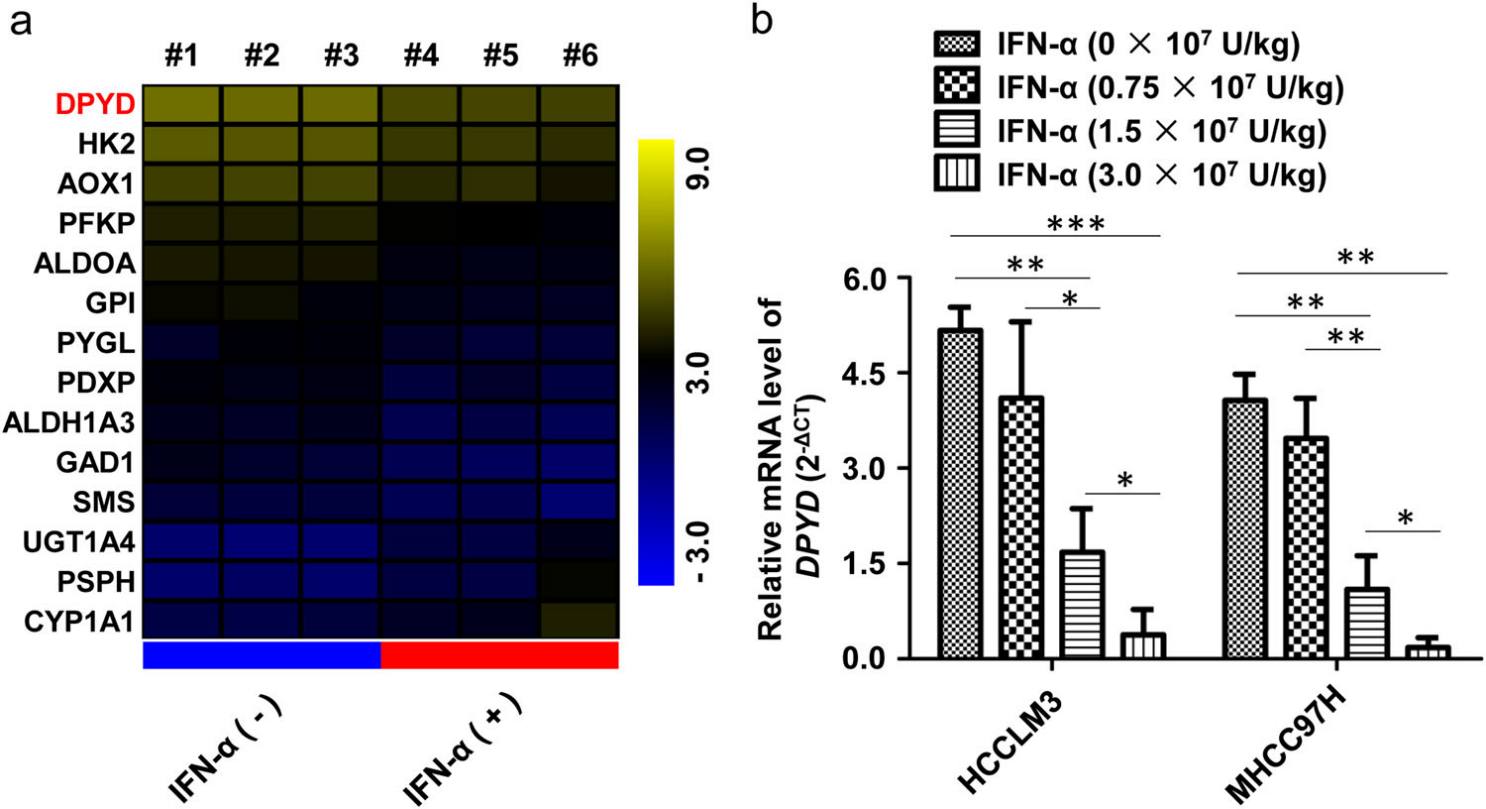
Identification of DPYD as a therapeutic target of IFN-α **a** HCCLM3 orthotopic mouse models were successfully established, and then treated with or without IFN-α (3 × 10^7^ U/kg/day) for 35 days. Altered metabolic gene profiling of these two groups was analyzed by RNA sequencing, and the top 10% of statistically differently expressed genes (*P* < 0.001) were shown by heat map. **b** The lead gene, DPYD, was dose-dependently downregulated by IFN-α both in HCC samples from either HCCLM3 or MHCC97H orthotopic mouse models by qRT-PCR analysis. Data, mean  ±S.D. *P < 0.05, **P < 0.01, and ***P < 0.001

Previously, we have found that IFN-α can inhibit tumor metastasis in nude mice bearing human HCC xenografts with high metastatic potential^4^. To identify the molecular biomarkers that were predictive for the response to IFN-α, we analyzed gene expression profiles by RNA sequencing in HCC samples from HCCLM3 mouse model with or without IFN-α treatment (3 × 10^7^ U/kg/day). Notably, DPYD, which plays a rate-limiting role in pyrimidine metabolism, was identified as the leading gene that was obviously downregulated in IFN-α-targeted molecules correlated with metabolism (Fig. 1a). Subsequently, we treated MHCC97H and HCCLM3 mouse models with increasing concentrations of IFN-α (0.75 × 10^7^, 1.5 × 10^7^, and 3 × 10^7^ U/kg/day) for 35 consecutive days^4^. Our data showed that IFN-α could dose-dependently downregulate the mRNA expression of DPYD in HCC samples from both MHCC97H and HCCLM3 mice (*P* < 0.05; Fig. 1b), suggesting that DPYD was a therapeutic target of IFN-α. Consistently, we found that the protein levels of DPYD in the samples from IFN-α-treated mice were also significantly downregulated compared to the mice without IFN-α treatment (*P* < 0.05; Supplementary Fig. S1). Together, these results indicated that DPYD might participate in IFN-α-inhibited HCC metastasis, thus we focused on the roles and mechanisms of DPYD in driving HCC metastasis.

### DPYD expression level is elevated in a HCC cell line panel

**Fig. 2:**
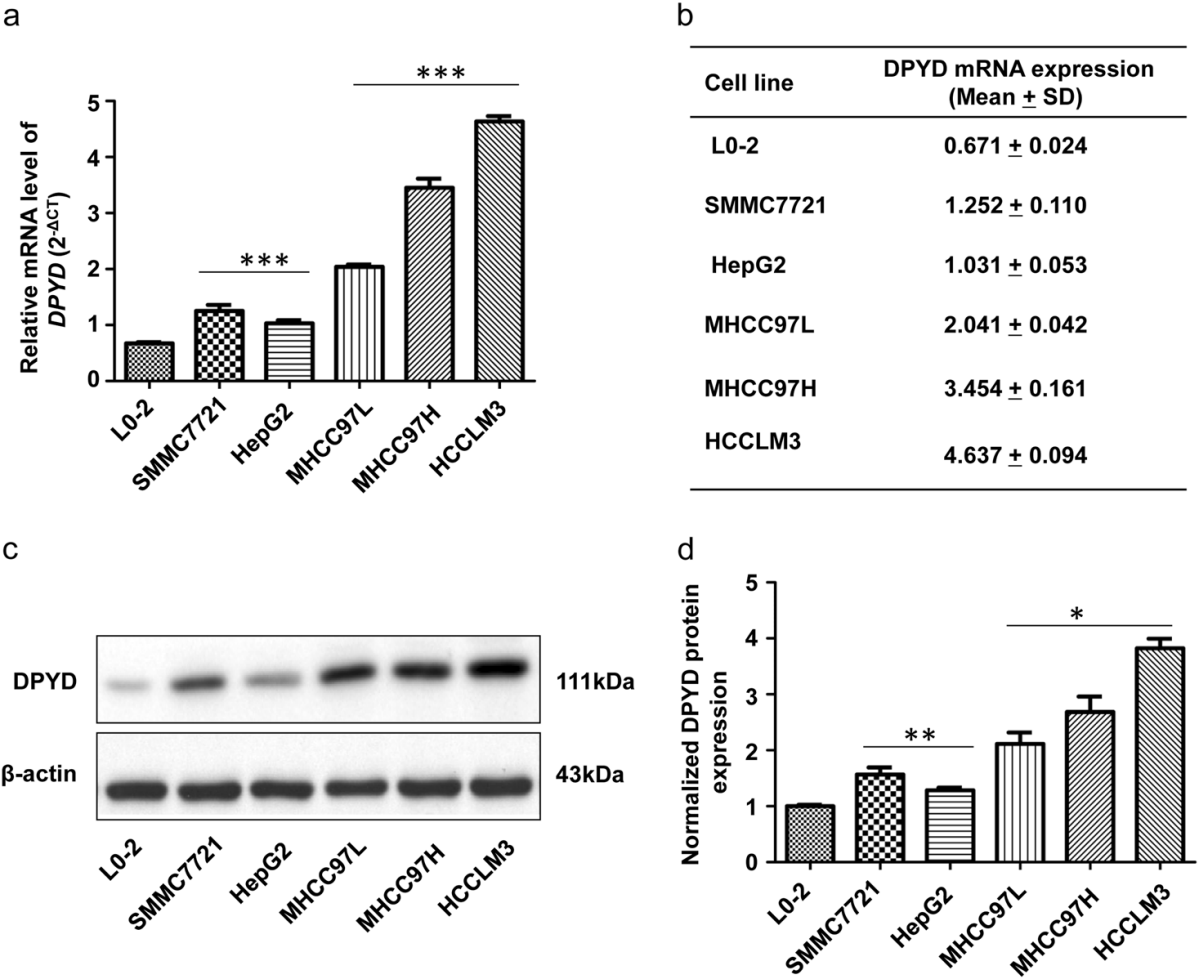
DPYD expression is upregulated in various human HCC cell lines The mRNA and protein expression of DPYD in five HCC cell lines (SMMC7721, HepG2, MHCC97L, MHCC97H, and HCCLM3) were compared with a nontransformed hepatic cell line (L0-2). **a**, **b** qRT-PCR analysis confirmed that the mRNA expression of DPYD in HCC cell lines is upregulated compared to hepatic cell line (L0-2). The DPYD mRNA expression was significantly increased in the HCC cell lines with high metastatic potentials (MHCC97L, MHCC97H, and HCCLM3) than those with low metastatic potentials (SMMC7721 and HepG2). Data, mean ± S.D. ***P < 0.001. **c**, **d** Western blot analysis showed a significant increase of DPYD in the HCC cells lines. The increased levels of DPYD protein were observed in the HCC cell lines with high metastatic potentials (MHCC97L, MHCC97H, and HCCLM3) compared to those with low metastatic potentials (SMMC7721 and HepG2). Data, mean ± S.D. *P < 0.05, **P < 0.01

To elucidate the exact role of DPYD in HCC metastasis, we firstly analyzed the expression of DPYD in HCC cell lines with different metastatic potential. We initially detected the mRNA levels of DPYD in five HCC cell lines (SMMC7721, HepG2, MHCC97L, MHCC97H, and HCCLM3) and one nontransformed hepatic cell line (L0-2). Data showed that the mRNA expression of DPYD are upregulated in these HCC cell lines compared to the L0-2 cell line (Figs. 2a, b). Moreover, the mRNA expression of DPYD in high metastatic potential HCC cell lines (MHCC97L, MHCC97H, and HCCLM3) were significantly increased compared to those in low metastatic potential HCC cell lines (SMMC7721 and HepG2). Then, DPYD protein levels in these cell lines were also assessed by western blot assay, which exhibited expression profile consistent with the mRNA expression profile (Figs. 2c, d). These results suggested that DPYD expression was closely associated with the metastatic potential of HCC cells.

### Expression of DPYD is upregulated in human HCC patients

**Fig. 3:**
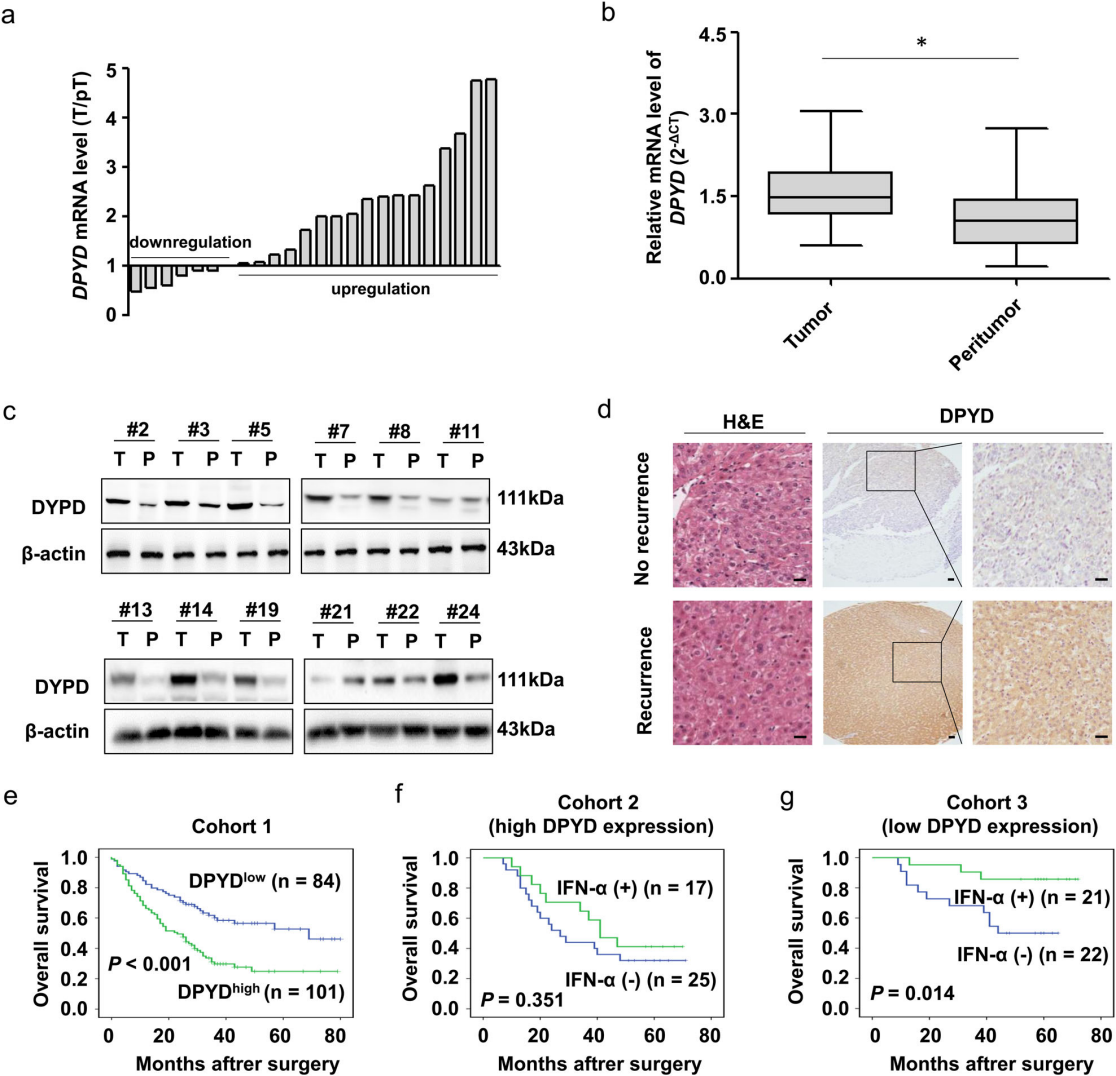
DPYD expression is upregulated in human HCC samples and correlated with HCC progression **a** Relative DPYD mRNA levels among peritumoral and tumoral tissues of HCC patients (*n* = 24) with distant metastasis after curative resection. **b** qRT-PCR analysis of DPYD levels in tumor samples (*n* = 24). The mRNA expression of DPYD is significantly higher in HCC tissues than in peritumor tissues. Data, mean ± S.D. *P < 0.05. **c** Western blot analysis showed the protein expression of DPYD in HCC tissues. **d** Expression of DPYD was examined by immunohistochemical staining analyses in 185 HCC specimens, and representative images are shown. (scale bar, 50 μm; original magnification for right panel: ×400). **e** Prognostic significance of DPYD in HCC patients was evaluated by Kaplan–Meier analysis. Upregulation of DPYD in HCC predicts lower overall survival times. **f**, **g** Association of DPYD expression with IFN-α therapeutic response in HCC patients. Kaplan–Meier analysis indicated that HCC patients with low DPYD expression have better response to IFN-α therapy

Given that DPYD expression is closely related to HCC metastasis, we firstly evaluated the expression of DPYD in a cohort of 24 HCC patients with metastasis after curative resection. Results showed that the mRNA levels of DPYD in HCC tissues were significantly upregulated compared to paired peritumor tissues (*P* < 0.05; Figs. 3a, b). Consistently, the results were also confirmed by western blot assay (Fig. 3c). Recurrence is a high-risk factor for poor prognosis in the progression of HCC after curative resection. To analyze the relationship between DPYD and recurrence, we further performed immunohistochemical analysis of DPYD in cohort 1 containing paired tumor and nontumor liver tissues from 185 HCC patients. We found that DPYD expression in HCC patients with recurrence were even higher than those without recurrence (Fig. 3d), which suggested that DPYD might play a potential role in HCC progression.

### DPYD predicts prognosis and response to IFN-α in HCC

To evaluate the clinical significance of DPYD in HCC, we then examined the relevance of DPYD expression and the clinicopathological features using immunohistochemical staining in cohort 1 containing 185 HCC patients underwent curative resection (Supplementary Table S1). Interestingly, elevated DPYD expression was identified to be closely associated with larger tumor size (*P* = 0.004), tumor recurrence (*P* < 0.001), and advanced tumor node metastasis (TNM) stage (*P* = 0.041). However, the other clinicopathologic features such as gender, age, HbsAg, HCV, liver cirrhosis, alpha-fetoprotein, tumor number, tumor encapsulation, or Edmondson grade exhibited no relationship with the expression of DPYD in HCC.

In this study, all 185 HCC patients in cohort 1 were divided into two groups: DPYD^low^ group (*n* = 84) and DPYD^high^ group (*n* = 101), with DPYD^high^ group accounting for 54.6% (101 of 185). Our data showed that the 1-year, 3-year, and 5-year OS rates in the whole cohort were 73.5%, 43.5%, 37.6%, respectively. Notably, the 1-year, 3-year, and 5-year OS rates in the DPYD^high^ group were markedly lower than those in the DPYD^low^ group (66.3% vs. 82.1%, 29.7% vs. 60.1%, 24.8% vs. 52.7%, respectively; Fig. 3e). Univariate and multivariate analyses demonstrated that DPYD was an independent prognostic factor for OS (HR = 1.556, *P* = 0.047; Supplementary Table S2). In addition, results showed that HCC patients with low DPYD expression had a significant improvement in survival when underwent IFN-α therapy (cohorts 2 and 3; *P* < 0.05; Figs. 3f, g). Collectively, our data clearly indicated that DPYD may have value as a predictive factor for prognosis of HCC patients and IFN-α therapeutic response.

### DPYD promotes metastatic potential of HCC cells in vivo and in vitro

**Fig. 4:**
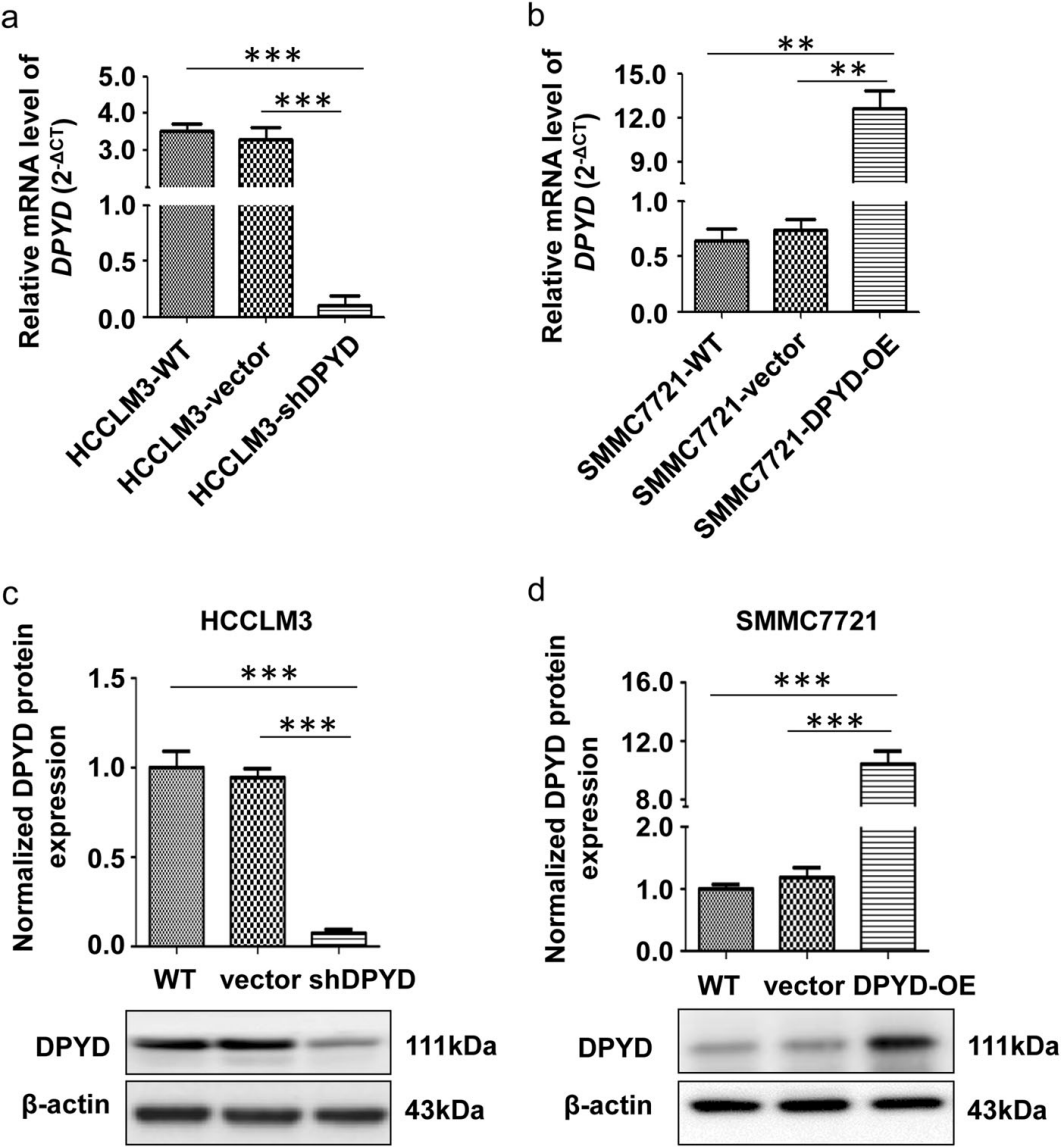
Establishment of different DPYD expression levels in various HCC cell lines qRT-PCR (**a**, **b**) and western blot (**c**, **d**) analysis showed the mRNA and protein expression of DPYD in SMMC7721 and HCCLM3 cells. Data, mean ± S.D. **P < 0.01, ***P < 0.001. *WT* wild type, *OE* overexpression

**Fig. 5:**
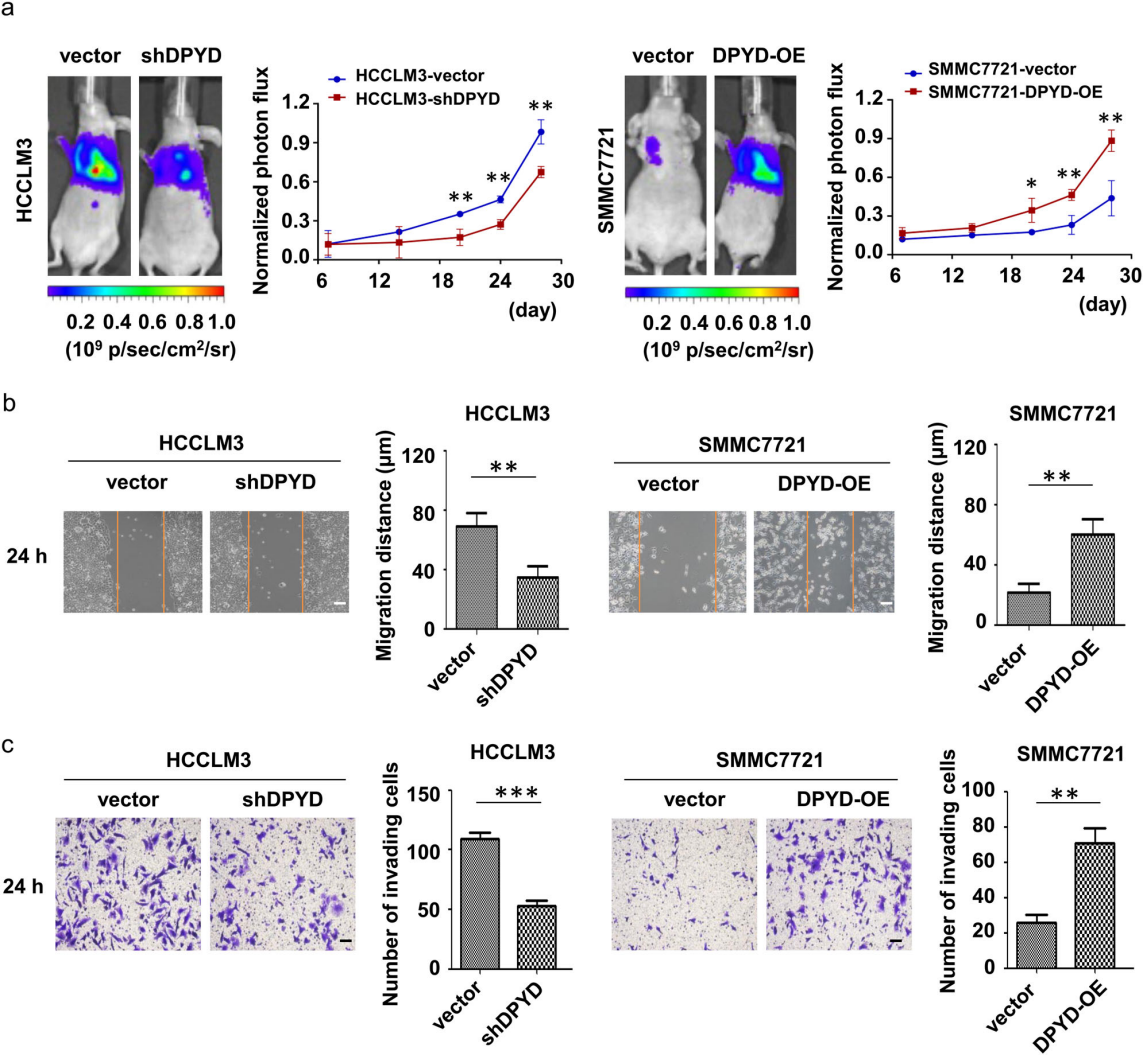
DPYD promotes metastatic potential of HCC cells in vivo and in vitro **a** In vivo imaging of lung metastasis of the four cell lines (SMMC7721-vector, SMMC7721-DPYD-OE, HCCLM3-vector, and HCCLM3-shDPYD) are shown by fluorescence in nude mice. The number of metastatic nodules in lung is significantly larger in the HCCLM3-vector group compared to the HCCLM3-shDPYD group from 20 days after tail vein injection. Similar results were observed in SMMC7721-DPYD-OE group compared to SMMC7721-vector group. *P < 0.05 and **P < 0.01. **b** The motility of the indicated cell lines in vitro was detected by scratch wound assay, and statistics are shown with a bar graph (original magnification: ×100; scale bar, 20 μm). **c** Invasive behavior in vitro was determined by transwell Matrigel invasion assay, and statistics are shown with a bar graph (original magnification: ×200; scale bar, 20 μm). Data, mean ± S.D., and representative of three independent experiments. **P < 0.01 and ***P < 0.001. *OE* overexpression

Since overexpression of DPYD in HCC independently correlated with poor prognosis, we further explored the molecular role of DPYD in the development of HCC using different HCC cell lines. DPYD expression in HCCLM3, a DPYD^high^ HCC cell line, was successfully downregulated (HCCLM3-shDPYD), and in SMMC7721, a DPYD^low^ HCC cell line, was stably upregulated (SMMC7721-DPYD-OE), compared with their parental cell lines (Figs. 4a–d). In this study, all cell lines for subsequent experiment were detected to rule out somatic mutations of DPYD. And then we assessed the in vivo and in vitro effects of these HCC cells on modulating HCC metastasis. Notably, our in vivo animal experiment showed that upregulation of DPYD in SMMC7721 cells (SMMC7721-DPYD-OE) increased the number of metastatic nodules in lung, while downregulation of DPYD in HCCLM3 cells (HCCLM3-shDPYD) reduced lung metastatic nodules, compared to their controls (Fig. 5a). Consistently, both the in vitro scratch assay and cell invasion assay demonstrated that inhibition of DPYD in HCCLM3 cells restrained the motility and invasiveness of these cells, whereas upregulation of DPYD in SMMC7721 cells enhanced these abilities, compared to their controls (*P* < 0.05; Figs. 5b, c). These studies clearly evidenced that DPYD could promote the motility and invasiveness of HCC cells in vivo and in vitro.

### DPYD enhances aggressiveness of HCC cells by promoting EMT

**Fig. 6:**
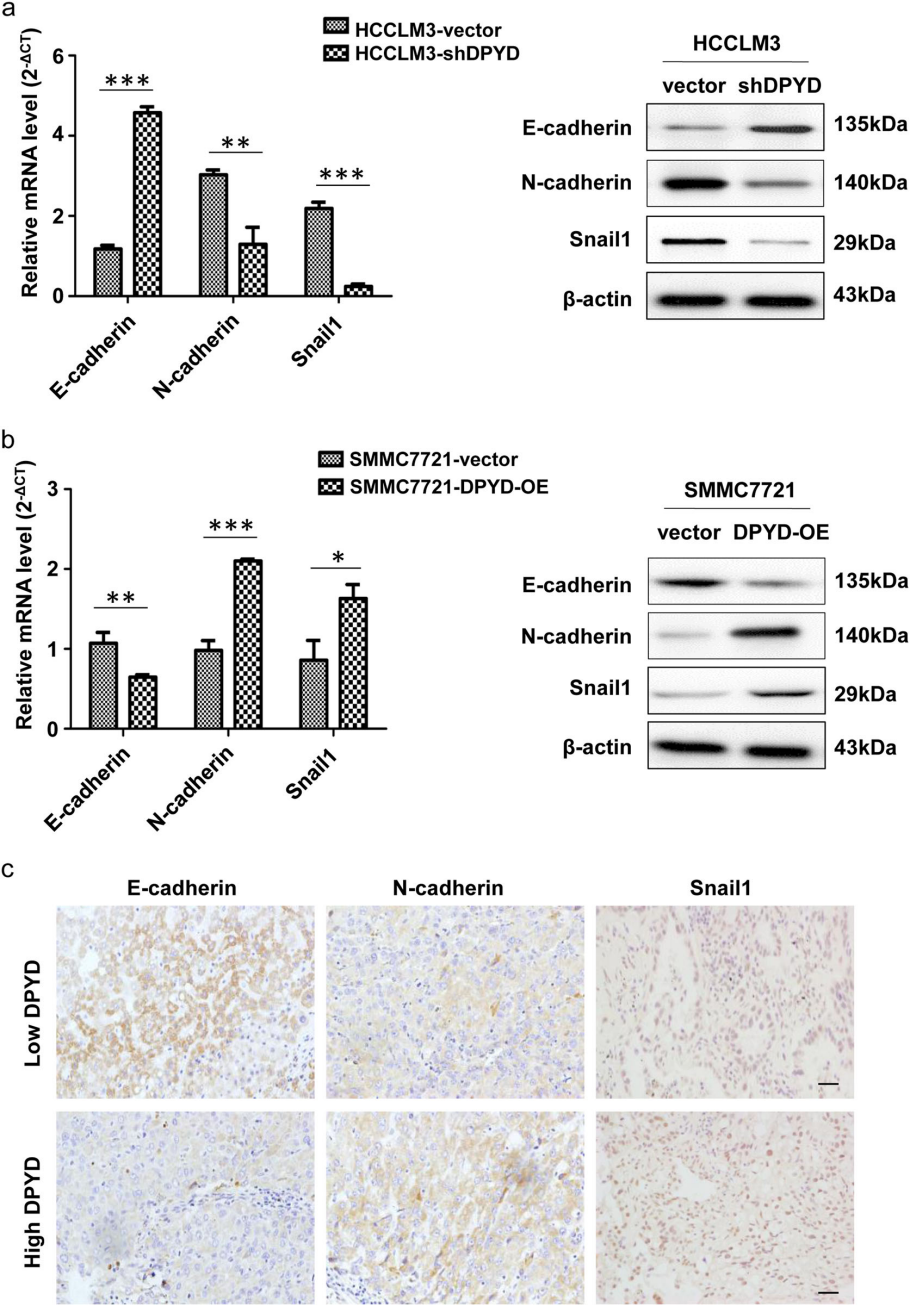
DPYD enhances aggressiveness of HCC cells by promoting EMT **a** The mRNA and protein expression changes of E-cadherin, N-cadherin, and Snail1 in SMMC7721-vector and SMMC7721-DPYD-OE cells. **b** qRT-PCR and western blot assays of these proteins in HCCLM3 cells with different DPYD expression. **c** Representative immunostaining images of EMT-related molecules from serial sections of human tumor samples (scale bar, 50 μm). Data represent the mean ± S.D. and are representative of three independent experiments. **P < 0.01, ***P < 0.001. *OE* overexpression

To confirm whether EMT is essential in DPYD-mediated aggressiveness of HCC cells, we further investigated expression changes of EMT markers in HCCLM3 and SMMC7721 cells with different DPYD expression (HCCLM3-vector, HCCLM3-shDPYD, SMMC7721-vector, and SMMC7721-DPYD-OE). Results showed that the mRNA and protein levels of E-cadherin were obviously upregulated in HCCLM3-shDPYD cells compared to HCCLM3-vector cells, while the levels of mesenchymal markers such as N-cadherin, ZEB1, and Snail1 were significantly downregulated (*P* < 0.05; Fig. 6a; Supplementary Fig. S2). Similar results were also observed in SMMC7721-vector cells compared to SMMC7721-DPYD-OE cells (*P* < 0.05; Fig. 6b; Supplementary Fig. S2). However, the activation of other EMT inducers such as MMP2, vimentin, and Twist1 displayed no significant changes (Supplementary Fig. S2). Consistently, immunohistochemistry (IHC) assay showed a similar phenomenon. Human HCC samples, which expressed high levels of DPYD, were found to express high levels of N-cadherin and Snail1, and low level of E-cadherin (Fig. 6c). These results strongly supported that DPYD could confer EMT-like traits on HCC cells, which might contribute to DPYD-induced HCC metastasis.

### DPYD facilitates EMT by suppressing the expression of target gene p38

**Fig. 7:**
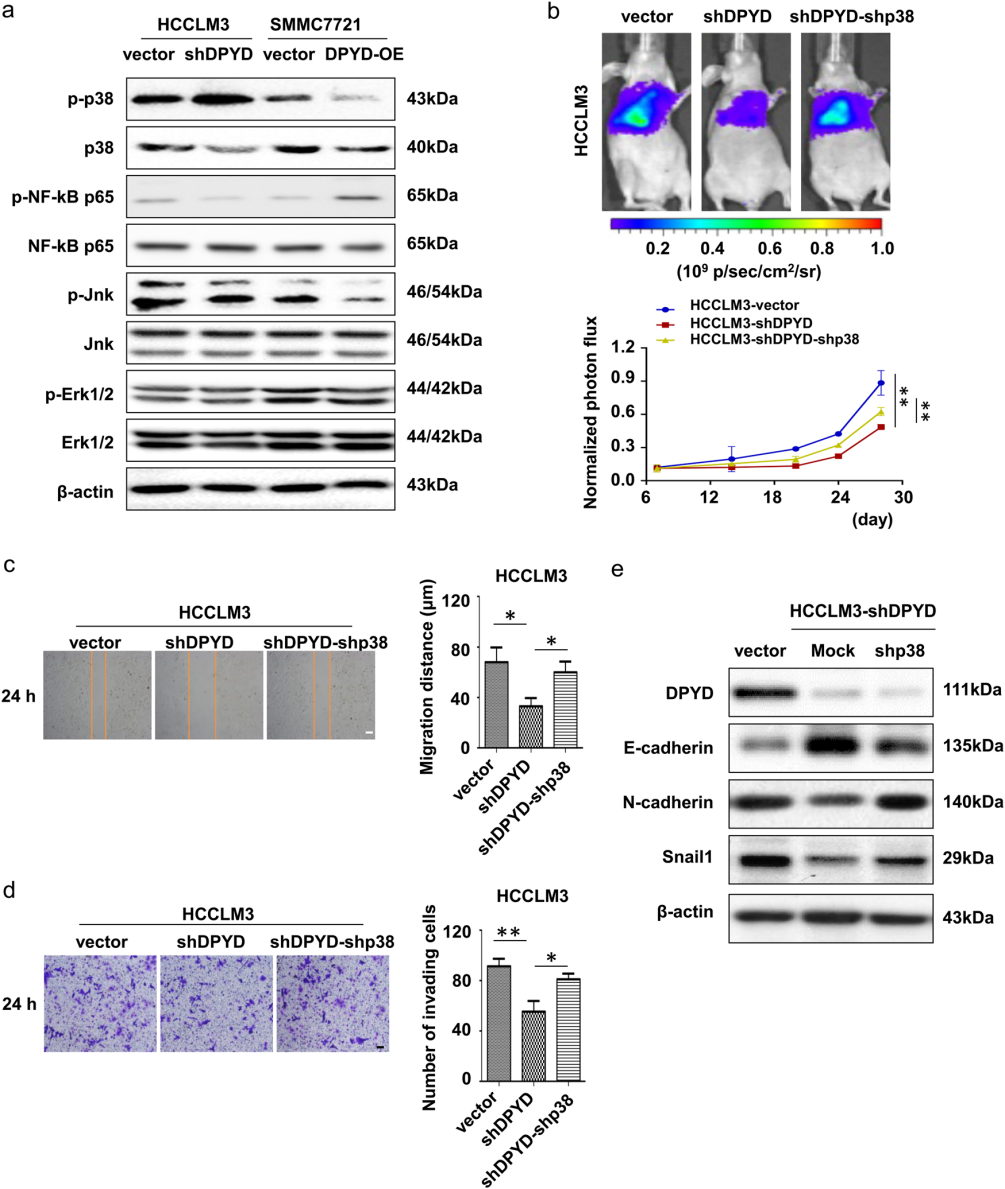
DPYD facilitates EMT by suppressing the expression of target gene p38 **a** The EMT-related signaling molecules were evaluated by western blot analysis in HCCLM3 and SMMC7721 cells with different DPYD expression. **b** Representative images of lung metastasis of HCC cell lines (HCCLM3-vector, HCCLM3-shDPYD, and HCCLM3-shDPYD-shp38) are shown by fluorescence in nude mice. The reduced number of metastatic nodules in lung was reversed by blockade of p38 in HCCLM3-shDPYD cells. Data, mean ± S.D. *P < 0.05, **P < 0.001. **c** Scratch wound assay showed that blockade of p38 in HCCLM3-shDPYD cells by p38-shRNA could significantly recover decreased cell migration due to DPYD deficiency (original magnification: ×100; scale bar, 20 μm). **d** Transwell invasion assay revealed that p38-shRNA treatment in HCCLM3-shDPYD cells markedly reversed the reduced cell invasive ability induced by DPYD deficiency (original magnification: ×200; scale bar, 20 μm). Data, mean ± S.D. *P < 0.05, **P < 0.001. **e** Expression changes of epithelial and mesenchymal markers at protein levels were detected by western blot analysis after p38 blockade in HCCLM3 cells. *OE* overexpression

To establish how DPYD affects EMT process, we evaluated the expression and phosphorylation of EMT-related molecules, such as p38, NF-κB p65, JNK, and Erk1/2, in HCCLM3 and SMMC7721 cells with different DPYD expression by western blot assay. Our results showed that the phosphorylation of p38 was significantly increased in HCCLM3-shDPYD cells but obviously reduced in SMMC7721-DPYD-OE cells, compared to their respective controls (Fig. 7a). In addition, we found that the phosphorylation of p65 was markedly declined in the cells with low DPYD expression (SMMC7721-vector and HCCLM3-shDPYD) but significantly enhanced in high DPYD expression cells (SMMC7721-DPYD-OE and HCCLM3-vector), which was inversely synchronized with the p38 activation. However, no other EMT-related signaling such as JNK and Erk1/2 was detected to be obviously changed according to the different expression levels of DPYD in HCC cells.

To verify the role of p38 in DPYD-mediated EMT process, the activation of p38 was blockaded using p38-shRNA in HCCLM3-shDPYD cells, which exhibited increased p38 phosphorylation due to DPYD deficiency. Our in vivo results showed that the reduced lung metastatic nodules in HCCLM3-shDPYD group could be reversed by the inhibition of p38 activation (*P* < 0.05; Fig. 7b). Moreover, in vitro functional assays demonstrated that, after the treatment of p38-shRNA, the reduced migratory and invasive abilities in HCCLM3-shDPYD cells were rescued compared to their respective controls (*P* < 0.05; Figs. 7c, d). Furthermore, western blot assay showed that upregulated E-cadherin expression in HCCLM3-shDPYD cells was reversed when treated with p38-shRNA or p38-inhibitor SB203580. In contrast, decreased mesenchymal markers such as N-cadherin and Snail1 in HCCLM3-shDPYD cells were partly recovered by p38-shRNA or SB203580 treatment (Fig. 7e; Supplementary Fig. S3). Evidently, our data certified that p38 plays a critical role in DPYD-mediated EMT in HCC cells.

### DPYD induces EMT via the p38/NF-κB/Snail1 signaling pathway

**Fig. 8:**
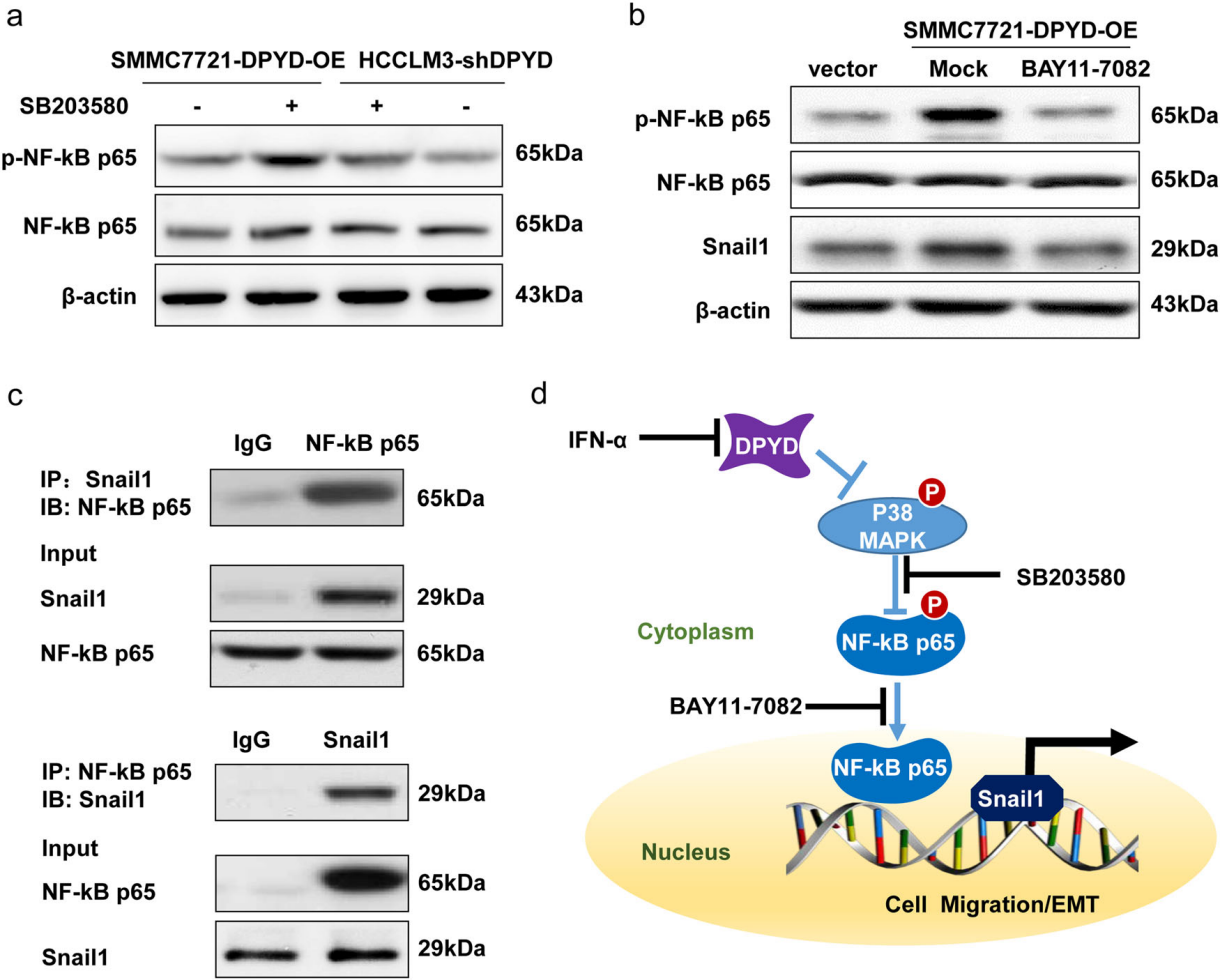
DPYD regulates EMT via the p38/NF-κB/Snail1 signaling pathway **a** Western blot assay showed that the expression of phosphorylated NF-κB p65 was regulated by p38 activity both in SMMC7721 and HCCLM3 cells when treated with p38 inhibitor SB203580. **b** Western blot assay showed simultaneous upregulation of Snail1 and NF-κB p65 phosphorylation in SMMC7721 cells with high DPYD expression. The activity of Snail1 was regulated by the level of NF-κB p65 phosphorylation in SMMC7721 cells when treated with p65 inhibitor BAY11-7082. **c** Co-immunoprecipitation assay indicated that NF-κB p65 formed a complex with Snail1 in SMMC7721 cells. **d** Schematic depiction of the mechanism underlying DPYD-mediated HCC metastasis in HCC cells. *OE* overexpression, *IgG* immunoglobulin G

It is increasingly appreciated that NF-κB is responsible for the process of tumor cells EMT^16,17^. In the above experiments, we confirmed that the activity of p38 had an inverse parallel with p65 phosphorylation (Fig. 7a). Thus, we wondered whether p38 is a negative regulator of p65 phosphorylation in DPYD-mediated EMT process. To identify this hypothesis, we inhibited p38 activity in SMMC7721-DPYD-OE and HCCLM3-shDPYD cells using p38-inhibitor SB203580. Results showed that the enhanced activity of p65 in SMMC7721-DPYD-OE cells, which had high DPYD expression, could further be strengthened by SB203580 treatment, and this process was actually due to the blockade of p38 activation (Fig. 8a). In contrast, the reduced phosphorylation of p65 in HCCLM3 cells with low DPYD expression could be reversed by the inhibition of p38 activation. Collectively, these results indicated that the phosphorylation of p65 mediated by DPYD is negatively regulated by p38.

Given that the activation of Snail1, a transcriptional driver of EMT, was altered simultaneously with p65 phosphorylation in different HCCLM3 cells (Fig. 7), we wondered whether p65 phosphorylation regulate Snail1 activity. As expected, our results showed that the increased Snail1 expression in SMMC7721-DPYD-OE cells, which had high DPYD expression, could markedly be reversed by p65-inhibitor BAY11-7082, indicating that the activity of Snail1 was regulated by p65 phosphorylation in HCC cells (Fig. 8b). Moreover, immunoprecipitation analysis indicated that after the activation of p65, p65 could form a complex with Snail1 (Fig. 8c), and then promote Snail1 transcriptional activity, suggesting that the activation of Snail1 in HCC EMT process needs activation and nuclear translocation of p65. Thus, the alteration of Snail1 activity, which in concordance with different DPYD levels in HCC cells, could be mediated by p65 phosphorylation. Together, these results demonstrated that DPYD facilitate EMT of HCC through p38/NF-κB/Snail1 signaling. Consistently, our data evidenced that IFN-α could restrain DPYD-induced aggressiveness of HCC cells by inhibiting EMT (Supplementary Fig. S4). Collectively, our data showed that IFN-α downregulated DPYD expression during EMT by targeting the p38/NF-κB/Snail1 pathway to inhibit HCC metastasis (Fig. 8d).

## Discussion

Aberrant metabolic alterations contribute to tumor progression, and attracted interest as a potential therapeutic target. However, few studies reported the adaptations of cellular metabolism for tumor when treated with IFN-α. In this study, the novel finding is that DPYD, a rate-limiting enzyme of pyrimidine metabolism, acted as a therapeutic target of IFN-α in HCC. Our data showed that DPYD is a potential tumor promoter that is distinctly upregulated, and potently promotes aggressiveness of HCC by regulating EMT process.

We found that the mRNA expression of DPYD is higher in HCC tissues than in paired peritumor tissues from HCC patients with metastasis after curative resection. Consistently, this phenomenon has also been observed in these HCC samples by western blot analysis. Thus, we wonder whether DPYD is responsible for HCC development. Interestingly, our clinical data further confirmed this possibility. By analyzing a large cohort of HCC samples, we found that HCC patients with recurrence in general exhibited higher DPYD expression than those without recurrence. Moreover, the data showed that the relative DPYD levels were positively associated with poor prognosis hallmarks of HCC, including larger tumor size, tumor recurrence, and advanced TNM stage. Furthermore, DPYD was an independent prognostic factor for overall survival (OS) times after curative resection. In this study, HCC patients with low expression of DPYD generally had better IFN-α therapeutic response. Thus, we believed that DPYD is predictive for risk prognostication in HCC development, and might act as a candidate prognostic biomarker.

The role of DPYD in the development of HCC is not clear. In this study, we found that DPYD promoted HCC metastasis, and was associated with EMT process. First, the mRNA and protein levels of DPYD were positively correlated with the increased metastatic abilities of HCC cell lines (Fig. 2). Second, the in vivo and in vitro experiments evidenced that upregulation of DPYD in HCC cells markedly enhanced the motility and invasiveness of HCC cells, whereas knockdown of DPYD inhibited these processes (Fig. 5). Third, upregulation of DPYD enabled HCC cells to acquire mesenchymal-like traits (Fig. 6). Indeed, EMT-related markers are predictors for increased invasion, metastasis, and poor prognosis in some human tumor types^18^. Collectively, it is reasonable to believe that DPYD could promote HCC metastasis through, at least partially, induction of EMT.

EMT is a program whereby epithelial-derived tumor cells acquire invasive and metastatic traits, and maintain high-grade malignancy^19^. Inhibition of EMT program may keep these cells stay in a lower-grade malignancy, and potentially reducing metastasis^20^. However, until now advances in inhibitors for the transcriptional drivers of EMT still remain a challenge^21,22^. Thus, the exploration of new targets in EMT process, which could potentially be intervened by small molecules, might provide a new strategy for cancer therapy. In this study, we firstly verified that DPYD could dose-dependently be downregulated by IFN-α in mice bearing human HCC xenografts, and is essential in the induction of EMT. Then, by western blot screening and subsequent confirmatory assays, we identified that p38 is a novel target gene of DPYD in HCC, and could regulate NF-κB/Snail1 signaling in DPYD-induced EMT (Figs. 7 and 8)^23^, which is in concordance with the effects in other cell lines as previously reported^24–26^. However, the other EMT markers, such as vimentin, MMP2, and Twist1, were not observed to be involved in the DPYD-mediated EMT in HCC. This discrepancy is probably due to the heterogeneity of the induced mesenchymal characteristics in the development of EMT^27,28^. Taken together, our findings link the activity of DPYD to IFN-α therapy, which might provide a new insight in the treatment of HCC.

Notably, increasing molecules were reported to function as a double-edged sword for cancer cell survival and progression^29–32^. As for DPYD, we and other groups identified it as a promoter of tumor development, and high DPYD expression was closely associated with poor patient prognosis^9–13^. Conversely, several studies found that high DPYD expression in tumor is a significant good prognostic factor^33,34^. Reconciling these discrepancies is difficult. One possibility is that the epidemiologic features and research approaches might affect DPYD expression profile^35^. Another possibility is that the function of DPYD might alter in the development of HCC^36^, which in accordance with the notion that some molecules might exhibit either suppressor or promoter activity in different tumor stages^37^. Surely, further studies are needed to investigate the underlying mechanisms regulating DPYD function.

In summary, our study implies DPYD as a potential target of IFN-α in a subset of HCC, and suggests that DPYD might be a novel biomarker for predicting patient prognosis.

## Materials and methods

### Patients and follow-up

Tumor samples and paired peritumor tissues (*n* = 24) were collected from the specimens of HCC patients after surgical resected at Fudan University Shanghai Cancer Center (Shanghai, PR China). Cohort 1 (185 HCC patients), cohort 2 (42 HCC patients), and cohort 3 (43 HCC patients) from the Liver Cancer Institute, Zhong Shan hospital, Fudan University (Shanghai, PR China) were randomly enrolled in this study. The paraffin-embedded tumor tissues from these patients were performed for tissue microarray (TMA) analyses.

All enrolled patients have not received preoperative anticancer treatment, and also have not underwent 5-fluorouracil-based chemotherapy. Preoperative liver function was evaluated according to the Child-Pugh scoring system. Tumor stage was assessed by the 2010 International Union Against Cancer TNM classification system. Tumor differentiation was determined by the classification proposed by Edmondson and Steiner. OS was defined as the dates between surgery and death, or between surgery and the last observation point. The data of patients alive were censored at the date of the last follow-up^38^. Prior patient consent and ethical approval from the Research Ethics Committee of Fudan University Shanghai Cancer Center were obtained. The follow-up procedures were performed as described previously^39^.

### Cell lines and transfection

Five HCC cell lines (SMMC7721, HepG2, MHCC97L, MHCC97H, and HCCLM3) and one nontransformed hepatic cell line (L0-2) were used in this study. L0-2 and HepG2 were purchased from American Type Culture Collection (ATCC, Manassas, VA). SMMC7721 was established by the Second Military Medicine College (Shanghai, China), and MHCC97L, MHCC97H, and HCCLM3 were established at the Liver Cancer Institute of Zhongshan Hospital (Shanghai, China).

The short hairpin RNA (shRNA)-mediated stable silencing technique was applied to evaluate the functional role of DPYD in HCC cells, which was previously described^40^. The designed target sequence for DPYD was cloned into the pLKO.1 TRC cloning vector (Supplementary Table S3). Lentiviral particles were constructed through co-transfection of the shRNA plasmid and the lentiviral enveloping and packaging plasmid (pMD2.G and psPAX2) into 293T cells. The HCC cells were transfected with the viral particles, and then selected with 2 mg/mL puromycin (P8833; Sigma-Aldrich). In this study, genomic analyses of DPYD were performed to rule out somatic mutations in HCC cells (Sangon Biotech, Shanghai).

### Tumor xenograft studies and imaging

The Institute Animal Research Committee at Fudan approved all animal handling. Male nude mice (4–6-weeks-old) were purchased from SLAC Laboratory Co., Ltd (Shanghai, China). The mice (*n* = 5 per treatment group) were intravenously injected to tail with 1 × 10^5^ HCC cells (200 µL in 2 min injections), stably knockdown DPYD, and luc-vector fusion proteins. The Luciferase signal intensities of the mice were determined using Caliper Life LifeSciences IVIS®Lumina/Living Image (Caliper LifeScience, Hopkinton, MA, USA) at 7, 14, 20, 24, and 28 days after tail vein injection. Briefly, after anesthetizing the mice, 200 μL of 15 mg/mL D-luciferin (Gold Biotechnology, USA) was injected intraperitoneally, and the whole body in vivo imaging analysis was performed for 30 s to 2 min, using in vivo imaging system (IVIS®Lumina).

### Quantitative real-time PCR and western blot assays

RNA isolation, cDNA synthesis, quantitative real-time PCR (qRT-PCR) reactions, and western blot assays were described in our previous study^41,42^. The primers and antibodies used in this study are listed in the Supplementary Tables S4 and S5.

### IHC analysis

The collected specimens of 185 patients with HCC were paraffin-embedded and stored at 4 °C. TMAs were constructed with these specimens, and IHC protocols were performed as described in our previous study^43^. The expression of DPYD in TMA was examined by IHC analysis, and the quantification of their expression levels was determined by the integrated optical density (IOD) as previously described^44^. The immunostaining intensities of DPYD were divided into two categories (DPYD^low^ expression and DPYD^high^ expression) by the median density of IOD, which was calculated as a cutoff value in the subsequent analyses.

### Cell migration assay

The scratch wound assay was used to detect the migration ability of HCC cells. The cells were incubated in a 24-well plate, and then wounded with a scratched line by 200 μL plastic pipette tip when grown to confluence. At 0 and 24 h, the migrating distance of the indicated cells at the wound front was calculated by an inverted microscope (Leica) in three randomly captured images.

### Cell matrigel invasion assay

Cell invasion assays were carried out by 24-well transwells (8 μm pore size; Corning, USA), which were precoated with Matrigel (Falcon354480; BD Biosciences, USA). Cells cultured in the upper chamber were suspended in serum-free medium, and serum-containing medium was placed in the lower chamber. After 24 h incubation, cells migrated to the bottom surface of the membrane were fixed and stained with Giemsa. The quantity of cells in five microscopic fields (magnification ×100) were counted and photographed in three independent experiments.

### Immunoprecipitation analysis

HCC cells were harvested and lysed with TNEN buffer supplemented with 1 mM NaF, 1 mM Na_2_VO_3_, 1 mM phenylmethanesulfonylfluoride, 1 μg/mL aprotonin, 1 μg/mL leupeptin, and 1 μg/mL pepstatin. Protein concentration was determined using a Bio-Rad assay (Bio-Rad, Hercules, CA, USA). The precleared cell lysates were incubated with primary mAb pre-absorbed protein A/G agarose beads (Roche, Indianapolis, IN, USA, 50% slurry equilibrated in phosphate-buffered saline). Immune complexes were collected after centrifugation, and the immunoprecipitates were separated by sodium dodecyl sulfate-polyacrylamide gel electrophoresis and transferred to polyvinylidene difluoride membranes. The membranes were incubated with primary antibodies, and then incubated with horseradish peroxidase-conjugated secondary antibodies (Cell Signaling, Danvers, MA, USA) and visualized with Enhanced Chemiluminescence reagent (Millipore, Billerica, MA, USA). The antibodies used were listed in Supplementary Table S5.

### Statistical analysis

Data were conducted with SPSS version 19.0 for Windows (IBM), and analyzed as previously described^45^. Pearson *Χ*^2^-test or Fisher exact test was used to compare categorical data. OS rate was calculated by the Kaplan–Meier method, and differences among groups were analyzed by the log-rank test. The Cox proportional hazards regression model was applied to perform univariate and multivariate analyses. All variables might be associated with OS were subjected to the multivariate Cox analysis with 0.2 level for entry into the model. Differences were considered statistically significant at *P* < 0.05.

## Supplementary information

Supplementary material
