# Supplementary material for: Dihydropyrimidine dehydrogenase predicts survival and response to interferon-α in hepatocellular carcinoma
Source: Cell Death Dis. 2018 Jan 22;9(2):69. doi: 10.1038/s41419-017-0098-0 (PMC5833634; doi:10.1038/s41419-017-0098-0)
Supplement: Supplementary file 1 — Supplementary material [file 41419_2017_98_MOESM1_ESM.docx]

**Supplementary data to:**

**Dihydropyrimidine dehydrogenase predicts survival and response to interferon-α in hepatocellular carcinoma**

Wei-Ping Zhu, Ze-Yang Liu, Yi-Ming Zhao, Xi-Gan He, Qi Pan, Ning Zhang, Jia-Min Zhou, Long-Rong Wang, Miao Wang, Di-Hua Zhan, De-Ning Ma, and Lu Wang

**Supplementary Figure S1.** Western blot analysis confirmed that the protein expression of DPYD could dose-dependently be downregulated by IFN-α both in HCCLM3 (a) and MHCC97H (b) orthotopic mice models. Data, mean + SD, and representative of three independent experiments. *P<0.05, **P<0.01, and ***P<0.001.

**
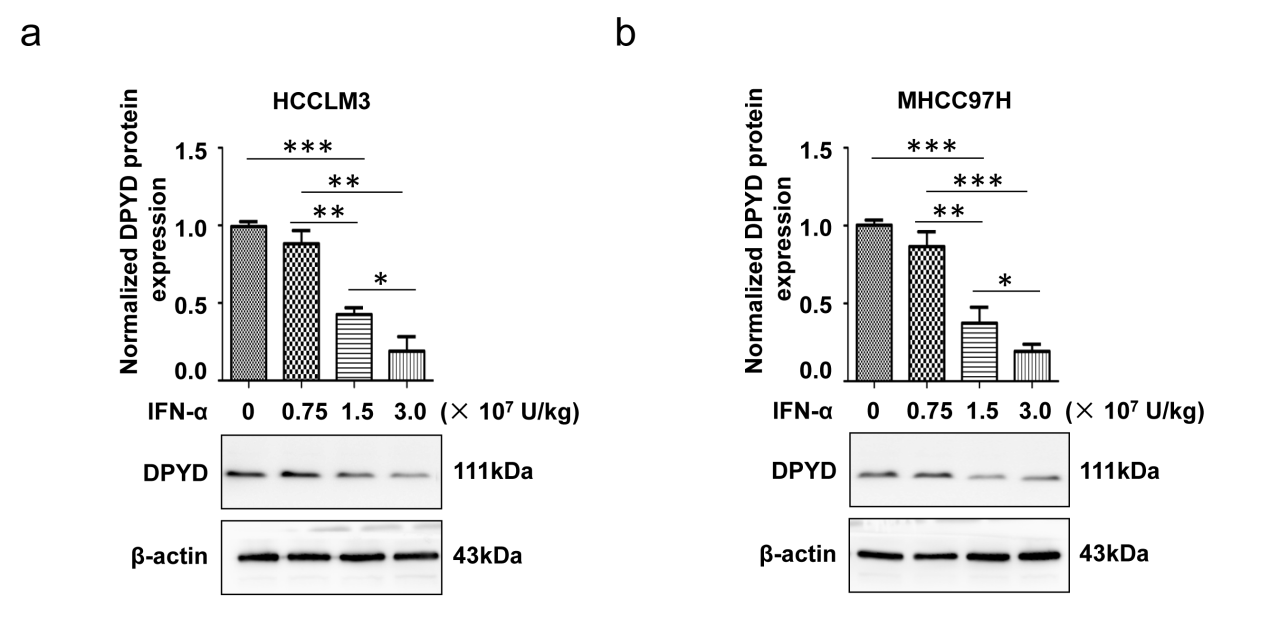
**

**Supplementary Figure S2.** qRT-PCR and western blot assays of ZEB1, MMP2, Vimentin, and Twist1 in HCCLM3 (a) and SMMC7721 (b) cells with different DPYD expression. **P<0.01.

**
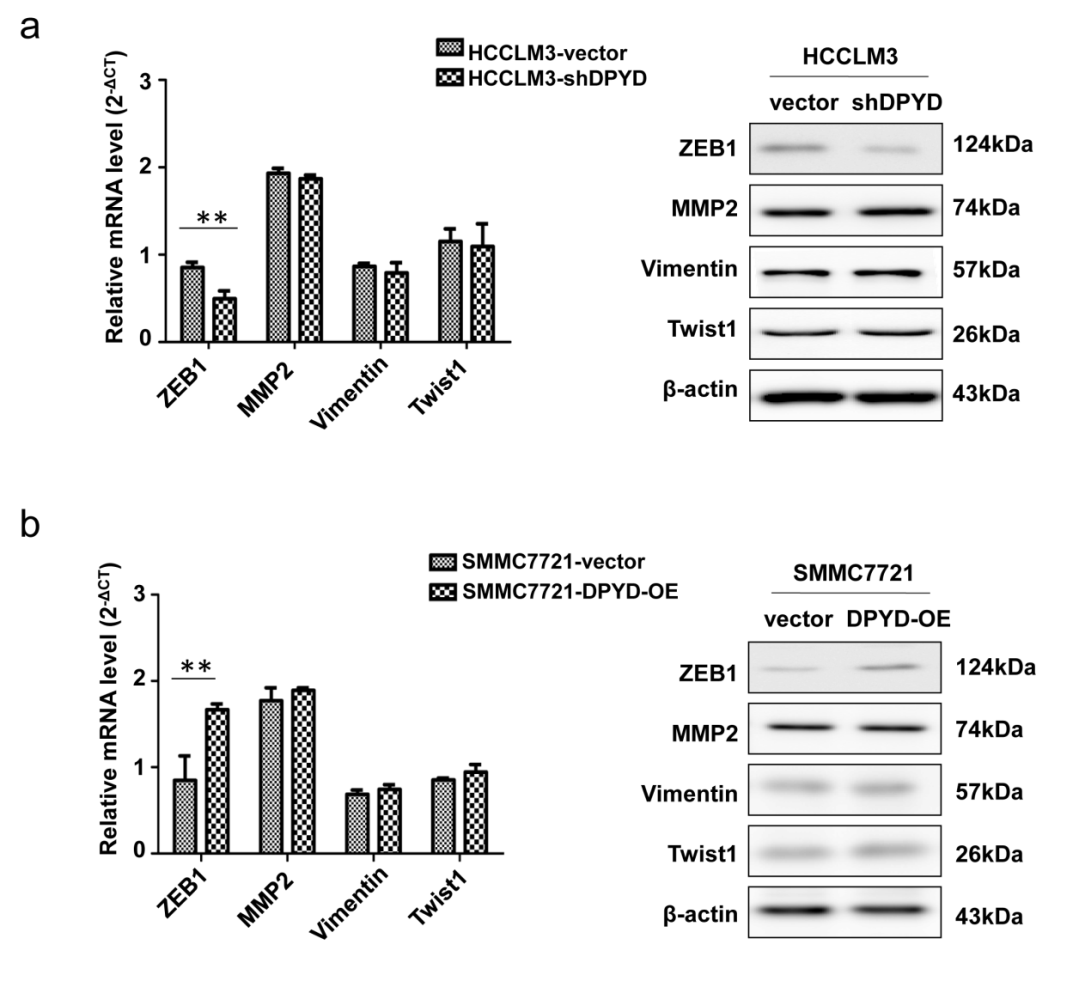
**

**Supplementary Figure S3.** The protein expression changes of DPYD, E-cadherin, N-cadherin, and Snail1 in HCCLM3-vector and HCCLM3-shDPYD cells when treated with p38-inhibitor SB203580.

**
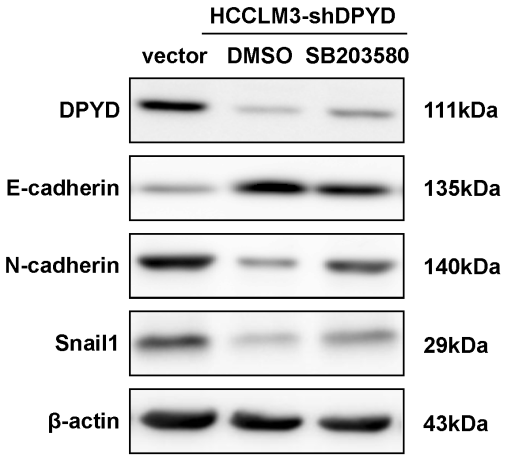
**

**Supplementary Figure S4.** IFN-α restrains DPYD-induced aggressiveness of HCC cells by inhibiting EMT. (a) The protein expression changes of E-cadherin, N-cadherin, and Snail1 in SMMC7721-DPYD-OE cells when treated with or without IFN-α (10^5^ U/mL). (b) The invasive behavior of the indicated cell lines *in vitro* were detected by transwell Matrigel invasion assay, and statistics are shown with a bar graph. (original magnification: ×100; scale bar, 20 μm). **P<0.01.

**
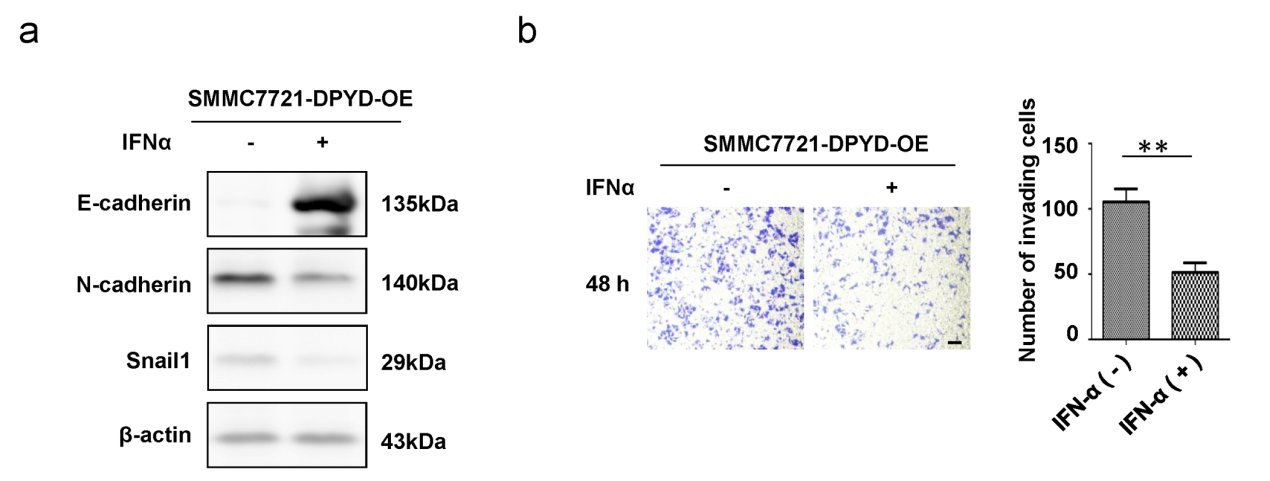
**

**Supplementary Table S1. Clinical characteristics of 185 HCC patients and correlation with DPYD staining.**

|  | **DPYD staining** | | |
| --- | --- | --- | --- |
| **Variables** | **Low(n=84 )**  **n(%)** | **High(n=101 )**  **n(%)** | ***P*** |
| **Gender** |  |  |  |
| Male | 70 (83.3) | 89 (88.1) | 0.351 |
| Female | 14 (16.7) | 12 (11.9) |  |
| **Age(year)** |  |  |  |
| ≤50 | 33 (39.3) | 37 (36.6) | 0.711 |
| >50 | 51 (60.7) | 64 (63.4) |  |
| **HBsAg** |  |  |  |
| Negative | 18 (21.4) | 17 (16.8) | 0.427 |
| Positive | 66 (78.6) | 84 (83.2) |  |
| **HCV** |  |  |  |
| Negative | 82 (97.6) | 96 (95.0) | 0.600 |
| Positive | 2 (2.4) | 5 (5.0) |  |
| **Liver cirrhosis** |  |  |  |
| No | 14 (16.7) | 13 (12.9) | 0.467 |
| Yes | 70 (83.3) | 88 (87.1) |  |
| **AFP(ng/ml)** |  |  |  |
| ≤ 20 | 32 (38.1) | 29 (28.7) | 0.177 |
| > 20 | 52 (61.9) | 72 (71.3) |  |
| **Tumor number** |  |  |  |
| Single | 79 (94.0) | 89 (88.1) | 0.165 |
| Multiple | 5 (6.0) | 12 (11.9) |  |
| **Tumor size(cm)** |  |  |  |
| ≤5 | 33 (39.3) | 20 (19.8) | **0.004** |
| >5cm | 51 (60.7) | 81 (80.2) |  |
| **Tumor encapsulation** |  |  |  |
| Complete | 43 (51.2) | 49 (48.5) | 0.717 |
| Imcomplete | 41 (48.8) | 52 (51.5) |  |
| **Metastasis/Recurrence** |  |  |  |
| No | 51 (60.7) | 30 (29.7) | **<0.001** |
| Yes | 33 (39.3) | 71 (70.3) |  |
| **Edmondson grade** |  |  |  |
| I-II | 61 (72.6) | 65 (64.4) | 0.230 |
| III-IV | 23 (27.4) | 36 (35.6) |  |
| **TNM stage** |  |  |  |
| I-II | 15 (17.9) | 8 (7.9) | **0.041** |
| III-IV | 69 (82.1) | 93 (92.1) |  |

Abbreviations: HBsAg, hepatitis B surface antigen; HCV, hepatitis C virus; AFP, alpha fetoprotein; TNM, tumor node metastasis; DPYD, dihydropyrimidine dehydrogenase.

**Supplementary Table S2. Univariate and Multivariate Analyses of Factors Associated with OS.**

|  | **Univariate Analyses** | |  | **Multivariate Analyses** | |
| --- | --- | --- | --- | --- | --- |
| **Variables^a^** | **HR (95% CI)** | ***P*** |  | **HR (95% CI)** | ***P*** |
| Gender (Male vs. Female) | 1.903 (0.991-3.651) | **.053** | | 1.823 (0.910-3.655) | .090 |
| Age (≤50 year vs. >50 year) | 1.110 (0.746-1.652) | .606 | |  | NA |
| HBsAg (Negative vs. Positive) | 1.103 (0.656-1.856) | .712 | |  | NA |
| HCV (Negative vs. Positive) | 1.546 (0.629-3.800) | .342 | |  | NA |
| Liver cirrhosis (No vs. Yes) | 1.599 (0.855-2.991) | **.142** | | 1.573 (0.804-3.079) | .186 |
| AFP (≤ 20 ng/ml vs. >20 ng/l) | 1.145 (0.755-1.738) | .523 | |  | NA |
| Tumor number (Single vs. Multiple) | 1.523 (0.834-2.780) | **.171** | | 1.625 (0.866-3.048) | .130 |
| Tumor size (≤5cm vs. >5cm) | 1.973 (1.239-3.143) | **.004** | | 1.925 (1.080-3.432) | **.026** |
| Tumor encapsulation(Complete vs. Imcomplete) | 1.152 (0.782-1.698) | .474 | |  | NA |
| Metastasis/Recurrence (No vs. Yes) | 2.235 (1.474-3.387) | **<.001** | | 2.265 (1.378-3.725) | **.001** |
| Edmondson grade (I-II vs. III-IV) | 1.616 (1.092-2.390) | **.016** | | 1.522 (1.023-2.264) | **.038** |
| TNM stage (I-II vs. III-IV) | 2.871 (1.328-6.205) | **.007** | | 0.851 (0.310-2.336) | .754 |
| DPYD (Low vs. High) | 2.192 (1.461-3.289) | **<.001** | | 1.556 (1.006-2.407) | **.047** |

^a^Variables were analyzed by Cox proportional hazards regression model.

Abbreviations: OS, overall survival; HR, hazard ratio; CI, confidence interval; HBsAg, hepatitis B surface antigen; HCV, hepatitis C virus; AFP, alpha fetoprotein; TNM, tumor node metastasis; DPYD, dihydropyrimidine dehydrogenase; NA, not adopted.

**Supplementary Table S3. Sequences of shRNA targeting DPYD**

|  | **Sequence (5’  . 3’)** |
| --- | --- |
| shRNA1 | TGCTGTTGACAGTGAGCGCGCAGATGCCCTGGAGTTAAATTAGTGAAGCCACAGATGTAATTTAACTCCAGGGCATCTGCTTGCCTACTGCCTCGGA |
| shRNA2 | TGCTGTTGACAGTGAGCGAGCCGGATTGAAGTTTATAAATTAGTGAAGCCACAGATGTAATTTATAAACTTCAATCCGGCCTGCCTACTGCCTCGGA |
| shRNA3 | TGCTGTTGACAGTGAGCGCTCGGTGAATGATGGAAAGCAATAGTGAAGCCACAGATGTATTGCTTTCCATCATTCACCGATTGCCTACTGCCTCGGA |

**Supplementary Table S4. Primers used in the study**

| **Primers for Real-time PCR** | **Sequence (5’** **. 3’)** | |
| --- | --- | --- |
| **Protein** |  |  |
| DPYD | F | CTTGGTCTGACTTGTGGAAT |
|  | R | TGGCAAAAAGTCTTTGGTA |
| E-cadherin | F | TTGCTACTGGAACAGGGACAC |
|  | R | CCCGTGTGTTAGTTCTGCTGT |
| N-cadherin | F | TTATCCTTGTGCTGATGTTTGTG |
|  | R | TCTTCTTCTCCTCCACCTTCTTC |
| Vimentin | F | CCTTGACATTGAGATTGCCACCTA |
|  | R | TCATCGTGATGCTGAGAAGTTTCG |
| Snail1 | F | TCCAGAGTTTACCTTCCAGCA |
|  | R | CTTTCCCACTGTCCTACTCTG |
| Twist1 | F | GTCCGCAGTCTTACGAGGAG |
|  | R | GTCTGAATCTTGCTCAGCTTGTC |
| ZEB1 | F | TGACATCACATAAATCAGGAAGAGA |
|  | R | GGGGTTGGCACTTGGTGGGATTA |
| MMP2 | F | ATGGAGGCGCTAATGGCCCGGG |
|  | R | ATCGCCATGCTCCCAGCGGCCAAAG |
| β-actin | F | CTGAGGACAAGCCACAAGATTA |
|  | R | ATCCACCAGAGTGAAAAGAACG |

**Supplementary Table S5. Antibodies used in the study**

| **Protein** | **Usage** | **Antibody** |
| --- | --- | --- |
| DPYD | WB, IHC | Ab54797, Abcam |
| E-cadherin | WB, IHC | 3195,CST |
| N-cadherin | WB, IHC | 14215,CST |
| Vimentin | WB, IHC | 5741,CST |
| Snail1 | WB, IHC, IP | 3879,CST |
| Twist1 | WB, IHC | ab50887, Abcam |
| ZEB1 | WB, IHC | 3396,CST |
| MMP2 | WB, IHC | 4022,CST |
| P38 | WB, IHC | 8690,CST |
| NF-κB p65 | WB, IHC, IP | 8242,CST |
| SAPK/JNK | WB, IHC | 9252,CST |
| Erk1/2 | WB, IHC | 4695,CST |
| β-actin | WB, IHC | 3700,CST |

Abbreviations: WB, western blot; IHC, immunohistochemistry; IP, immunoprecipitation
